# Two Distinct Subtypes Revealed in Blood Transcriptome of Breast Cancer Patients With an Unsupervised Analysis

**DOI:** 10.3389/fonc.2019.00985

**Published:** 2019-10-01

**Authors:** Wenlong Ming, Hui Xie, Zixi Hu, Yuanyuan Chen, Yanhui Zhu, Yunfei Bai, Hongde Liu, Xiao Sun, Yun Liu, Wanjun Gu

**Affiliations:** ^1^State Key Laboratory of Bioelectronics, School of Biological Science and Medical Engineering, Southeast University, Nanjing, China; ^2^The First Affiliated Hospital of Nanjing Medical University, Nanjing, China

**Keywords:** peripheral blood mononuclear cells, immune gene signature, unsupervised analysis, breast cancer subtype, breast cancer survival

## Abstract

**Background:** Breast cancer (BC) is a highly heterogeneous cancer. The interaction between immune system and BC is complex, widespread yet unclear. In this study, we aimed to reveal the heterogeneity of host systemic immune response to BC and understand the possible mechanisms that may drive the heterogeneity using transcriptomic data from peripheral blood mononuclear cells (PBMCs).

**Methods:** Transcriptome-wide gene expressions of PBMCs in 33 BC patients were generated by RNA sequencing. An unsupervised clustering algorithm was employed to discover PBMC transcriptome subtypes among BC patients. Association analysis between PBMC subtypes and age, clinical stage, abundance of immune cells, and other clinical factors was performed to understand the underlying biological processes that may drive this heterogeneity. Immune gene signature identification and *in silico* survival analysis were performed to investigate the potential clinical implications of these PBMC subtypes. The findings were validated using the whole blood transcriptomes of an independent cohort.

**Results:** We observed that established BC subtypes were not associated with PBMC gene expression profiles. Instead, we discovered and validated two new BC subtypes using PBMC transcriptome, which have distinct immune cell proportions, especially for lymphocytes (*P* = 5.22 × 10^−12^) and neutrophils (*P* = 1.13 × 10^−14^). Enrichment analysis of differentially expressed genes revealed that these two subtypes had distinct patterns of immune responses, including osteoclast differentiation and interleukin-10 signaling pathway. We developed two immune gene signatures that can differentiate these two BC PBMC subtypes. Further analysis suggested they had the ability to predict the clinical outcome of BC patients.

**Conclusions:** PBMC transcriptome profiles can classify BC patients into two distinct subtypes. These two subtypes are mainly shaped by different immune cell abundance, which may have implications on clinical outcomes.

## Introduction

Breast cancer (BC) is now the most frequently diagnosed cancer and the sixth leading cause of cancer-related death among Chinese women (1). To gain better outcomes, the early diagnosis, prognosis and treatment monitoring are critically important (1). However, BC is well-known as a highly heterogeneous malignant tumor, both molecularly and histologically. At present, BC has been classified into five intrinsic molecular subtypes, including luminal-A, luminal-B, HER2-enriched, basal-like, and normal-like (2–5). Each subtype has distinct gene expression profiles, which is associated with cancer prognosis, disease progression, cancer metastasis, and therapeutic resistance (2–5). Based on several clinical and pathological factors, such as estrogen receptor (ER), progesterone receptor (PR), and human epidermal growth factor receptor 2 (HER2) status, BC is routinely divided into several subtypes in clinical implementation (6, 7). These clinical classifications are frequently used to guide the treatment of BC patients (6, 7).

Although genetic and epigenetic changes are the key causes of BC, both the innate and adaptive immune system may play substantial roles in BC progression and metastasis as well (8). The presence of cancer cells can activate different immune cells to undergo various phenotypic and functional changes, and eventually kill cancer cells or promote the proliferation of cancer cells (9, 10). Several studies have attempted to detect the presence of cancers by profiling the gene expression in peripheral blood mononuclear cells (PBMCs) from BC patients (11–14) and some other malignant tumors (15, 16). They have proposed several PBMC gene expression signatures that can significantly differentiate cancer patients from healthy controls (12, 13, 15, 16). Furthermore, expression profiles of several immune-related genes in PMBCs from BC patients can predict the relapse of triple negative BC (11, 14). These findings indicated that transcriptomic analysis of peripheral blood immune cells (PBMCs) might be a practical way to evaluate the host systemic immune responses against cancer cells. Notably, this is especially valuable, since the collection of blood samples is non-invasive and convenient as compared to the sampling of tumor tissues (11). However, the human immune system is substantially variable (17). A wide range of factors, such as age, sex, genetic background, and some environmental influences, can perturb and shape the blood transcriptome (17). The relationship between immune system and BC is intricate, and many unanswered questions remain (8, 18). Among them, one of the most important issues is to explore the heterogeneity of blood transcriptome of BC patients and the clinical relevance of this heterogeneity.

In this study, we aimed to reveal the heterogeneity of host systemic immune response to BC and understand the possible mechanisms that drive the heterogeneity. First, we measured the transcriptome-wide gene expressions in PBMC samples from 33 BC patients using RNA sequencing (RNA-seq), and correlated the gene expression profiles with known clinical classifications. Next, we performed an unsupervised cluster analysis on PBMC expressions to reveal the heterogeneity among BC patients and *de novo* classified BC patients with distinct host response patterns. Then, we validated the PBMC subtypes in an independent BC dataset. Furthermore, we investigated possible clinical factors that may be related to the PBMC subtypes of BC patients, including age, clinical stages and the abundance of immune cells. Finally, we explored the potential of using PBMC gene signatures to predict the clinical outcome of BC patients.

## Materials and Methods

### Overview of Patient Cohorts

In this study, we recruited 33 BC patients from the First Affiliated Hospital of Nanjing Medical University, between July and September 2017, as a discovery cohort. All patients participated anonymously in consideration of privacy and security concerns. The detailed baseline demographic information of the discovery cohort is listed in Table 1. In IHC subtyping, ER positive, HER2 negative, high PR expression (more than 20%) and low Ki-67 expression (<14%) patients were defined as luminal-A subtype. ER positive, HER2 negative, low PR expression (<20%) or high Ki-67 expression (more than 14%) patients were defined as luminal-B subtype. Additionally, ER positive and HER2 positive patients were defined as luminal-B subtype as well (19). Upon recruitment, fresh peripheral blood samples were collected before clinical treatment. To validate the unsupervised classification of PBMC transcriptome in BC patients, we also downloaded the whole blood gene expression data and the clinical features of another BC cohort from European Genome-phenome Archive (accession number: EGAD00010001063) (20). This validation cohort includes 173 BC patients in the Norwegian Women and Cancer Study (21). The whole blood transcriptome was quantified by Illumina Human AWG-6 and HT12, including microarray expression data for 16,782 genes (21). The baseline characteristics of BC patients in the validation cohort are shown in Additional File 1. To estimate the proportion of tumor infiltrated lymphocytes (TILs) in BC, we also downloaded the transcriptome level gene expression data of 173 tumor tissue samples for all patients in the validation cohort from European Genome-phenome Archive (accession number: EGAD00010001064) (21).

**Table 1:** Demographics of BC patients in the discovery cohort.

**Characteristic**	**All patients** **(*n* = 33)**
Age (y)^*^	51.3 (24–77)
**Menopausal status**	
Premenopausal	0
Postmenopausal	0
Not available	33
**Histological type**	
Invasive ductal carcinoma	30
Invasive lobular carcinoma	1
Intraductal carcinoma	2
**ER status**	
Positive	22
Negative	11
**PR status**	
Positive	19
Negative	14
**HER2 status**	
Positive	19
Negative	14
**Ki-67 status**	
Less than 20%	9
More than 20%	24
**IHC-based subtypes**	
Luminal-A	16
Luminal-B	6
HER2-positive	3
Triple negative	8
**Pathological stage**	
Stage I	16
Stage II	11
Stage III	3
Not available	3

*Unless otherwise indicated, data are number of patients*.

* *Data for continuous variables are means, with ranges in parentheses*.

### Isolation of Total RNA From PBMC and RNA-Seq

PBMC samples of 33 BC patients in the discovery cohort were isolated from whole blood applying Ficoll-Paque Premium (*GE Healthcare*, IL, USA) according to the manufacturer's instructions. Total RNA was extracted from PBMC using TRIzol reagent (*Invitrogen*, CA, USA) and purified with the mirVana RNA Isolation Kit (*Ambion*, Massachusetts, USA) in accordance with the manufacturer's protocol. The purity and concentration of RNA were determined from OD260/280 readings using NanoDrop ND-1000. RNA integrity was determined by 1% formaldehyde denaturing gel electrophoresis. Only RNA extracts with RNA integrity number values >6 were used for further experiments. The isolated RNAs were immediately frozen in liquid nitrogen, and stored at −80°C. RNA-seq libraries were constructed by *Ovation* human FFPE RNA-seq library systems (*NuGEN Technologies*, CA, USA) and sequenced on *Illumina* HiSeq X Ten platform (*Illumina*, CA, USA) using paired-end 150 bp runs.

### RNA-Seq Data Analysis

RNA-seq reads were aligned to human genome 19 by *HISAT2* (22), quantified by *featureCounts* (23) and assembled by *StringTie* (24). The expression level of genes was quantified in forms of both counts data and normalized FPKM (fragments per kilobase of exon per million reads mapped). In total, expression values of 57,773 unique genes in PBMC samples of BC patients in the discovery cohort were measured. Considering the different types of gene expression profiles in the discovery and validation cohorts, *GLM* in *DESeq2* (25) was used to perform the differential gene expression analysis for RNA-seq data, while linear models in *limma* (26) was used for microarray data. Genes with a fold change in expression level of <0.25 or >4.0 and FDR-corrected *P* < 0.01 were identified as significant differentially expressed genes (DEGs). The annotation and enrichment visualization of DEGs were accomplished using *Metascape* (http://metascape.org) (27) and *Reactome* pathway database (https://reactome.org/) (28). The Gene Ontology (GO) terms, Kyoto Encyclopedia of Genes and Genomes (*KEGG*) pathways and *Reactome* pathways with a *P* < 1 × 10^−5^ in the enrichment analysis were retained.

### Discovery and Validation of the PBMC Subtypes

We used unsupervised consensus clustering (29) to discover intrinsic PBMC subtypes in the discovery and validation cohorts, respectively. The consensus clustering is a resampling-based method to represent the consensus across multiple runs of a clustering algorithm and to assess the stability of the discovered clusters (29). The method, which is robust and insensitive to the initial conditions, has been widely used to identify biologically meaningful clusters (29). In detail, we first selected the top 5,000 variable genes measured by median absolute deviation as the most informative genes for class detection. Then, we performed a bootstrap procedure with 80% item resampling and 80% gene resampling on the PBMC gene expression profiles 10,000 times using the agglomerative hierarchical clustering algorithm with the *Spearman* distance metric. We selected the optimal number of clusters that corresponds to the most stable consensus matrices and the most unambiguous cluster assignments across permuted clustering runs by varying the number of clusters from 2 to 10 (29). This process determined the optimal number of intrinsic unsupervised clusters defined by PBMC transcriptome in the discovery cohort. To validate the result, we implemented the same procedure on the validation cohort. In addition, we used in-group proportion (IGP) statistical analysis (30) to demonstrate the existence of the clusters in the validation cohort and evaluate the reproducibility of the clusters derived from consensus clustering in the two independent cohorts. IGP provides a quantitative approach to measure the similarity between the clusters. IGP will be 100% if the clusters are identical between two datasets and will be 0% conversely. Due to the different types of expression values in the two datasets, we normalized the expression data by Z-score prior to the IGP statistical analysis. The consensus clustering and IGP analysis were performed in R (https://www.r-project.org/) (31).

### Estimation of the Abundance of Major Immune Cells Using Gene Expression Profiles

We used *CIBERSORT* (https://cibersort.stanford.edu/) (32) with the *LM22* signature gene matrix (32) to characterize the proportion of immune cells in the PBMC sample of each BC patient in both discovery and validation cohorts. *CIBERSORT* is able to accurately estimate cell composition of complex tissues from their gene expression profiles, including the immune cells in human PBMC samples (32). We obtained the proportion of seven major immune cell types, including lymphocytes (consisting of all types of B cells, T cells, and NK cells), monocytes, macrophages (consisting of M0, M1, M2 macrophages), dendritic cells (consisting of resting and activated dendritic cells), mast cells (consisting of resting and activated mast cells), eosinophils and neutrophils. All subsequent analysis of immune cell proportions in this study was based on the estimation of these seven major cell types.

### Survival Analysis

We identified the immune-related gene signatures using their expression in PBMC samples. To explore the implication of the immune-related gene signatures on the patient's survival, we used *Kaplan-Meier-plotter* (http://www.kmplot.com/) (33) to perform *in silico* survival analysis. *Kaplan-Meier-plotter* is able to assess the effect of 54,000 genes on cancer survival in 21 cancer types, including BC, using their expression profiles in the tumor tissue (33).

### Statistical Analysis

To compare the clinical characteristics, cell proportions and established subtypes between clusters in both cohorts, we performed the *Fisher's* exact test or *Pearson's* chi-squared test for categorical variables and the *Student's t*-test for continuous variables. All statistical analysis were performed in R (https://www.r-project.org/) (31).

## Results

### Established Clinical Classifications Cannot Explain PBMC Expression Heterogeneity Among BC Patients

First, we explored the heterogeneity of PBMC transcriptome among the BC patients. We observed that a substantial number of genes varied significantly in expression in PBMC samples of the BC patients in both cohorts (Additional File 2). To explain this variation, we projected the PBMC transcriptome differences among BC patient groups onto known clinical classification. In the discovery cohort, the status of three immunohistochemistry (IHC) markers was available for each patient. We classified BC patients using all three IHC markers' status and compared the gene expression of BC patients with different ER, PR, and HER2 status. No significant difference was found between BC patients with different IHC markers' status (Additional File 3).

In the validation cohort, only the status of ER and HER2 was available. We tested the expression differences in patients with ER and HER2 status. Again, we found no significant difference (Additional File 4). In addition, gene expression profile of the matched tumor tissue is available for each patient in the validation cohort. With the expression data, we further classified the patients in the validation cohort into PAM50 subtypes (2) and investigated the PBMC transcriptome variations among these patient groups. The result indicated that PBMC gene expression in the BC patients with different PAM50 subtypes are statistically similar (Additional File 4). All these results suggested that the established known subtypes based on IHC marker and PAM50 were not associated with PBMC gene expression in BC patients.

### Identification and Validation for PBMC Transcriptome-Based Subtypes for BC Patients

**Figure 1:**
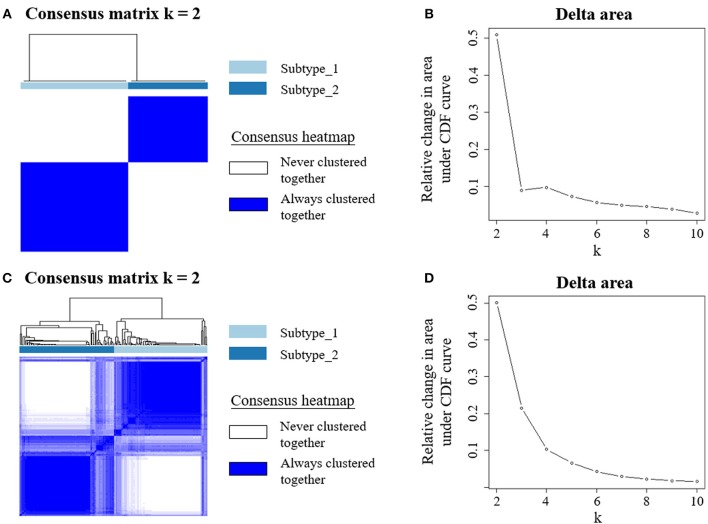
Unsupervised consensus clustering of PBMC transcriptome subtypes. Consensus matrix heatmaps for the chosen optimal cluster number (*k* = 2) for the discovery **(A)** and validation cohorts **(C)**, respectively. Rows and columns are patient samples and consensus matrix values range from 0 (never clustered together) to 1 (always clustered together). The dendrogram above the heatmap illustrates the ordering of patient samples in 2 clusters. The relative change in area under the cumulative distribution function (CDF) curves when cluster number varying from *k* to *k*+1 for discovery **(B)** and validation data **(D)**. The range of *k* changed from 2 to 10, and the optimal *k* = 2.

Next, we employed an unsupervised clustering algorithm to classify the BC patients into *de novo* groups based on their heterogeneity of systemic immune response to BC. We selected the top 5,000 genes with the highest median absolute deviation of expression values in the discovery cohort, and classified BC patients into two clusters, subtype_1 and subtype_2 (Figure 1A), using the consensus clustering algorithm (29). The 2-cluster solution corresponded to the largest cluster number that induced the least incremental change in the area under the cumulative distribution function (CDF) curves while keeping the maximal consensus within clusters and the minimal rate of ambiguity in cluster assignments (Figure 1B). Finally, subtype_1 includes 19 patients (58%), while subtype_2 includes 14 patients (42%).

To confirm this *de novo* classification, we independently applied the same analysis procedure (29) on the validation dataset, which is whole blood transcriptome data. Interestingly, we observed that the samples in the validation cohort were also clustered into two optimal clusters, which is very similar to that identified in the discovery dataset (Figures 1C,D). We evaluated the reproducibility of the two PBMC subtypes across the discovery and validation cohorts using in-group proportion (IGP) statistic (30). The IGP values are 89.8 and 75.3% for subtype_1 and subtype_2, respectively, indicating that both subtypes had high consistency between the two cohorts. This suggested that these two PBMC transcriptome subtypes are robust across different BC cohorts.

**Figure 2:**
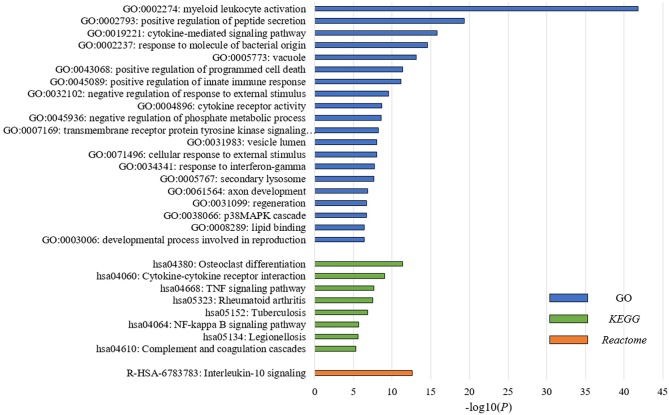
PBMC subtypes shared distinct molecular pathways and immune response patterns. GO terms, *KEGG* pathways, and *Reactome* pathways with a *P* < 1 × 10^−5^ in the enrichment analysis are displayed. We observed distinct immune patterns between the two PBMC subtypes. These distinct patterns cover the whole process of host immune response to tumor, including the activation of immune cells, the regulation and response of innate and adaptive immune system, and the production of some specific antibodies.

To understand the underlying biological mechanisms that differ in these two PBMC subtypes, we performed differential gene expression analysis using *DESeq2* (25). We observed 1,988 DEGs between these two subtypes in the discovery cohort. In enrichment analysis for the DEGs, the top 20 significantly enriched GO terms are related to immune regulation (Figure 2). Among them, myeloid leukocyte activation was the most significant GO term. Similarly, the enriched *KEGG* pathways and *Reactome* pathways (Figure 2) include osteoclast differentiation and interleukin-10 signaling, which associate to host immune response. The results suggested that the major differences between these two subtypes may be explained by their different immune responses to BC.

### PBMC Transcriptome Subtypes Are Distinct in Terms of Immune Cell Abundance

Then, we investigated possible clinical factors that relate to the two subtypes in the BC patients, including age, clinical stage, established BC subtype, blood immune cell abundance, and TILs. In the discovery cohort, there was no statistical difference between the two subtypes in terms of age, histological type or clinical stage (Table 2), or age, menopausal status or weight in the validation cohort (Table 3). Moreover, we found that the known established BC subtypes, including IHC marker status, IHC-based subtypes, and PAM50 intrinsic molecular subtypes, cannot account for the differences between PBMC transcriptome subtypes (Tables 2, 3), because both PBMC subtypes contained the BC patients with IHC marker status and PAM50 subtypes.

**Table 2:** Differences of established BC subtypes and clinical characteristics in PBMC subtypes in the discovery cohort.

**Clinical factors**	**Subtype_1** **(*n* = 19)**	**Subtype_2** **(*n* = 14)**	***P*-value**
**ER status**			1^a^
ER+ subtype	13	9	
ER– subtype	6	5	
**PR status**			1^a^
PR+ subtype	11	8	
PR- subtype	8	6	
**HER2 status**			0.723^a^
HER2+ subtype	10	9	
HER2– subtype	9	5	
**IHC-based subtype**			0.309^a^
Luminal-A	10	6	
Luminal-B	3	3	
HER2-positive	3	0	
Triple negative	3	5	
**Histological type**			0.496^a^
Invasive ductal carcinoma	16	14	
Invasive lobular carcinoma	1	0	
Intraductal carcinoma	2	0	
**Pathological stage**			0.169^a^
Stage I	10	6	
Stage II	4	7	
Stage III	3	0	
Age^*^(y)	47.9 (24–73)	55.9 (41–77)	0.052^b^

*Unless otherwise indicated, data are number of patients or the P-value of statistical test*.

a *P-value for the Fisher's exact test*.

b *P-value for the Student's t-test*.

* *Data for the continuous variables are means with ranges in parentheses*.

**Table 3:** Differences of established BC subtypes and clinical characteristics in PBMC subtypes in the validation cohort.

**Clinical factors**	**Subtype_1** **(*n* = 88)**	**Subtype_2** **(*n* = 85)**	***P*-value**
**ER status**			0.301^a^
ER+ subtype	68	71	
ER– subtype	20	14	
**HER2 status**			0.6973^a^
HER2+ subtype	18	22	
HER2– subtype	70	63	
**PAM50 molecular subtype**			0.656^a^
Luminal-A	24	24	
Luminal-B	21	22	
HER2-enriched	11	15	
Basal-like	15	14	
Normal-like	17	10	
**Menopausal status**			0.429^a^
Premenopausal	6	7	
Postmenopausal	74	64	
Weight^*^(kg)	72.9 (50–120)	70.2 (50–150)	0.219^b^
Age^*^(y)	56.7 (43–104)	55.9 (44–66)	0.445^b^

*Unless otherwise indicated, data are number of patients or the P-value of statistical test*.

a *P-value for Pearson chi-square test*.

b *P-value for Student's t-test*.

* *Data for continuous variables are means, with ranges in parentheses*.

Interestingly, we observed significant differences in proportion of lymphocytes (in the discovery cohort: *P* = 5.22 × 10^−12^; in the validation cohort: *P* = 5.80 × 10^−18^) and proportion of neutrophils (in the discovery cohort: *P* = 1.13 × 10^−14^; in the validation cohort: *P* = 1.86 × 10^−24^) between the two PBMC transcriptome-based subtypes (Table 4). Furthermore, we calculated the neutrophil-to-lymphocyte ratio (NLR), a common and stable hematological indicator that can reflect the inflammatory state of the body (34, 35). In comparing the NLR values between the two subtypes, we also observed a significant difference (in the discovery cohort: *P* = 6.60 × 10^−6^; in the validation cohort: *P* = 9.08 × 10^−21^). Other immune cells, such as the monocytes in the discovery cohort and the macrophages in the validation cohort, do not show a significant difference (Table 4).

**Table 4:** Differences of immune cell components in PBMC subtypes in the discovery and validation cohorts.

**Immune cell types**	**Discovery cohort** **(*n* = 33)**	**Validation cohort** **(*n* = 173)**
Lymphocytes	5.22 × 10^−12^*^^	5.80 × 10^−18^*^^
Monocytes	5.29 × 10^−5^*^^	0.509
Macrophages	0.579	0.00026^*^
Dendritic cells	0.001^*^	0.252
Mast cells	0.022	0.00076^*^
Eosinophils	0.399	0.166
Neutrophils	1.13 × 10^−14^*^^	1.86 × 10^−24^*^^

*Unless otherwise indicated, data are the P-value of Student's t-test*.

* *P < 0.01*.

**Figure 3:**
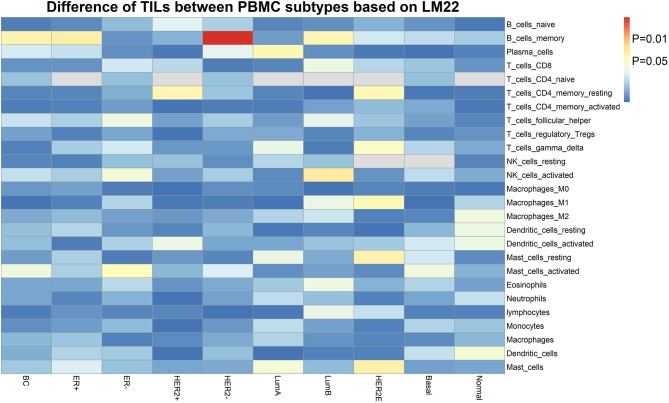
The heatmap of TIL differences between patients with two PBMC subtypes. Each row represents an immune cell type identified by *LM22*, and each column represents an established subtype of BC patients. The value of matrix is the *P*-value of the TIL difference (*Student's t*-test) between patients with PBMC subtype_1 and subtype_2.

Furthermore, we assessed the TIL differences in tumor tissue samples of patients with different PBMC transcriptome subtypes. We estimated the proportion of immune cells in the tumor tissue sample of each BC patient in the validation cohort, using *CIBERSORT* with the *LM22* signature (32). We found the tumor infiltration of memory B cells is statistically different in BC patients with two PBMC transcriptome subtypes (Figure 3), including all BC patients (*P* = 0.032), ER+ patients (*P* = 0.027), Luminal-B patients (*P* = 0.036) and HER2– patients (*P* = 0.0022). Additionally, memory resting CD4+ T cells is differentially infiltrated in cancer tissues of patients with different PBMC subtypes in HER2+ patients (*P* = 0.034) and HER2-enriched patients (*P* = 0.037).

These results suggested that the composition of immune cells in PBMCs and TILs in tumor tissues, rather than age, clinical stage, and known BC subtypes, are related to the heterogeneity of PBMC transcriptome in BC patients.

### PBMC Transcriptome Subtypes May Be Related to BC Survival

**Figure 4:**
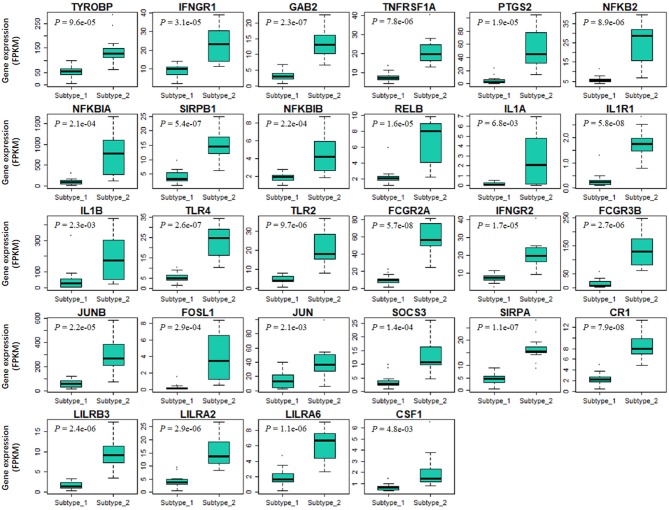
The expression values of 28 immune-related signature genes are significantly different in two PBMC subtypes. The 28-gene signature are derived from immune-related genes in osteoclast differentiation pathway. X-axis and Y-axis are the two PBMC subtypes and gene expression level normalized by FPKM, respectively. *P*-value is the result of the *Student's t*-test. All these genes are significantly low-expressed in subtype_1.

**Figure 5:**
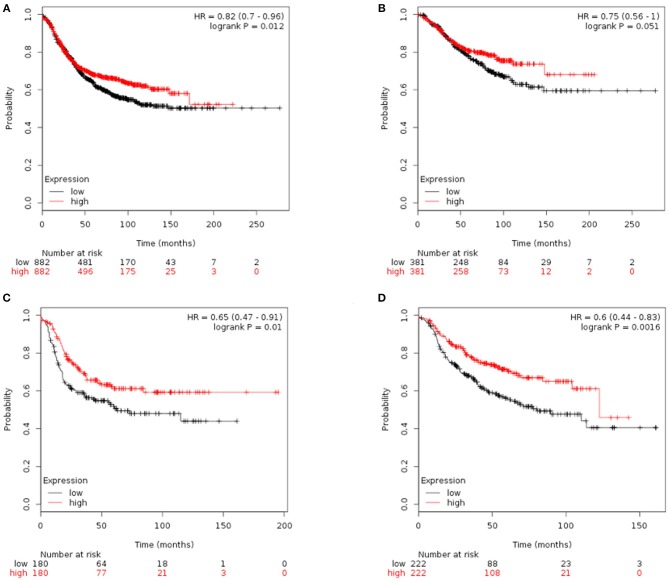
*Kaplan-Meier* curves of RFS stratified by the 28-gene signature. Prediction result of all subtypes of BC patients **(A)**, ER positive patients **(B)**, basal-like patients **(C)**, and clinical stage III patients **(D)**. The higher expression of signature genes in the tumor tissue corresponded to a lower risk of cancer recurrence and better survival rate.

Finally, we tried to explore the implications of the PBMC transcriptome heterogeneity on BC management. In the previous results, we found no difference in several available clinical characteristics between the two subtypes (Table 3). However, NLR, which is an indicator of the inflammation level, differed between the two subtypes. The inflammation level has important potential in predicting the clinical outcome of BC (36). We investigated if patients with different PBMC subtypes have different survival rate. Twenty-eight immune-related genes were identified in the pathway of osteoclast differentiation, which is the most enriched *KEGG* pathway (Table 5). Expression values of all the 28 genes were significantly higher in subtype_2 than in subtype_1 (Figure 4). Using *Kaplan-Meier-plotter* (33), we observed that the tissue expression values of the 28-gene signature had the ability to predict the clinical outcomes of all subtypes of BC patients (Figure 5A), as well as ER positive patients (Figure 5B), basal-like patients (Figure 5C) and clinical stage III patients (Figure 5D). The high expression of these 28 genes in tumor tissue, including IFNGR1, IFNGR2, IL1A, IL1B, TLR2, TLR4, FOSL1, and CSF1, associates with a lower risk of cancer recurrence and better survival rate in BC patients (Figure 5).

**Table 5:** Gene symbols of the 28-gene signature.

	**Gene symbol**
28-gene signature	TYROBP, IFNGR1, GAB2, TNFRSF1A, PTGS2, NFKB2, NFKBIA, SIRPB1, NFKBIB, RELB, IL1A, IL1R1, IL1B, TLR4, TLR2, FCGR2A, IFNGR2, FCGR3B, JUNB, FOSL1, JUN, SOCS3, SIRPA, CR1, LILRB3, LILRA2, LILRA6, CSF1

Furthermore, we repeated the analysis above and identified 16 immune-related genes in the most enriched *Reactome* pathway (Additional File 5). Similarly, 16 genes including IL1R2, CXCL1, CXCL8, PTGS2, IL1A, IL1RN, and CSF1 were highly expressed in the subtype_2 BC patients (Additional File 6). High expression of these genes in tumor tissue, were related to a low risk of recurrence and better survival rate in all subtypes of BC patients, ER positive patients, luminal-A patients, luminal-B patients and clinical stage III patients (Additional File 7). However, both gene signatures had no statistical power in differentiating the clinical outcomes of PR positive patients, HER2 positive patients, HER2-enriched patients, or other clinical stages BC patients (detailed in Additional Files 7, 8).

## Discussion

In this study, we revealed substantial heterogeneity of PBMC transcriptome in BC patients (Additional File 2) and identified two subtypes based on the PBMC gene expression profiles (Figure 1). Our results indicated that these two subtypes had distinct molecular pathways in host immune response and regulation (Figure 2). We observed that the PBMC-transcriptome based subtyping was a novel and independent classification for BC patients. The essential molecular basis of the subtyping reflects the interaction between host immune system and BC. We found that the proportion of immune cells in peripheral blood, especially lymphocytes and neutrophils, shaped the significant differences between the two subtypes (Table 4). Furthermore, two gene signatures that discriminates these two PBMC subtypes are able to predict the clinical outcomes of BC patients (Figure 5 and Additional File 7). Importantly, such subtyping is general and robust, since they were independently observed in both the discovery dataset and validation dataset. In the discovery dataset, we quantified PBMC transcriptome using RNA-seq technology, while the transcriptome data in the validation dataset was gene expression array (12). Although the quantification platform and source samples are different in these two datasets, the findings are consistent (Figure 1). However, a future study using a large prospective cohort will be highly helpful to validate these two PBMC subtypes in BC, since the sample size in the discovery cohort is relatively small.

Current clinical classifications did not reflect the heterogeneity of interactions between BC and host immune system (Tables 2, 3). This is consistent with several previous findings, suggesting that transcriptional fingerprint of BC subtypes is not the predominant signal in the patient's systemic immune response (14, 21). Thus, it was difficult to classify BC patients into classical BC subtypes using the PBMC expression profiles. The classification of the established BC subtypes was based on the expression of several important makers in tumor tissue, including ER, PR, and HER2 (6, 7). In contrast, PBMCs contains the major inflammatory or supportive cells, which are composed of the main stromal components of tumor microenvironment and govern the systemic inflammatory responses in human malignancies, including BC (37). Therefore, it was reasonable that PBMC transcriptome cannot mirror the different expression profiles in tissue samples among BC patients of different clinical subtypes. Instead, PBMC gene expression profiles might be useful for early diagnosis of human cancers, such as BC and colorectal cancer (11–13, 38).

In order to explore the heterogeneity of host systemic immune response to BC, we employed an unsupervised clustering algorithm to cluster BC patients using PBMC gene expression data, and revealed two distinct subtypes (Figure 1). Functional annotation and enrichment analysis displayed distinguishing immune patterns between the two subtypes (Figure 2). These distinct patterns covered the whole process of host immune response to tumor, including the activation of immune cells, the regulation and response of innate and adaptive immune system, and the production of some specific antibodies. Considering *KEGG* categorizes genes into meaningful biological pathways, which makes the interpretation more straightforward (39), we focused on the enriched *KEGG* pathways below. In our results, osteoclast differentiation, cytokine-cytokine receptor interaction and TNF signaling pathway were the top three *KEGG* pathways that had distinct expression patterns between the two subtypes. Osteoclasts are multinucleated cells of monocyte/macrophage origin that degrade bone matrix. The differentiation of osteoclasts is dependent on a tumor necrosis factor (TNF) family cytokine, receptor activator of nuclear factor (NF)-κB ligand (RANKL), as well as macrophage colony-stimulating factor (M-CSF) (40). BC frequently metastasizes to the skeleton, interfering with the normal bone remodeling process and inducing bone degradation (41, 42). Cytokines are highly inducible, secretory proteins that mediate intercellular communication in the immune system. Cytokine and cytokine receptor interaction are regarded as crucial aspects of inflammation and tumor immunology (43). Although the exact initiation process of BC is unknown, inflammation has been proposed as an important factor in tumor initiation, promotion, angiogenesis, and metastasis, in which cytokines are prominent players (44, 45). Moreover, many studies suggested that cytokines play an important role in the regulation of both induction and protection in BC (46, 47). TNF is a proinflammatory cytokine that plays a critical role in diverse cellular events, including cell proliferation, differentiation and apoptosis (48). TNF-α is an important inflammatory factor that acts as a master switch in establishing an intricate link between inflammation and cancer (48). A wide variety of evidence has pointed to a pivotal role of TNF-α in tumor proliferation, migration, invasion and angiogenesis, including BC (49, 50). These enriched pathways hinted that the different status of inflammation may partly explain the differences between PBMC transcriptome subtypes of BC patients, which may be related to BC metastasis.

The correlation of PBMC heterogeneity to BC metastasis is also confirmed by the differential analysis of immune cell proportions. Our results showed significant differences of the proportions of lymphocytes and neutrophils in the peripheral blood and the neutrophil-to-lymphocyte ratio (NLR) in the two subtypes (Table 4). The proportion of lymphocytes in subtype_1 was higher than that in subtype_2, whereas neutrophils were merely the major component of PBMCs in subtype 2. Several previous studies suggested that peripheral blood lymphocytes expressed abundant information about the interactions between the tumors and the host immune system, which are useful biomarkers for predicting the risk of cancer occurrence and recurrence (51, 52). Neutrophils, altering the local microenvironment by releasing inflammatory signals and promoting the formation of metastases, were considered as the main driving force of pulmonary metastatic colonization of BC cells (36, 53, 54). Neutrophils were also observed to be useful biomarkers for clinical BC diagnosis and prognosis assessment (36, 53, 54). Additionally, the pre-treatment NLR was a prognostic factor for BC (34, 35, 55, 56). A higher NLR was associated with poorer recurrence-free survival in BC patients (34, 35, 55, 56). In addition to immune cells that are circulating in the peripheral blood, BC patients with different PBMC transcriptome subtypes showed distinct TILs in tumor tissues (Figure 3). Although the precise role of tumor-infiltrating lymphocytes in cancer development and metastasis is not well-understood and remains controversial, accumulating evidences suggest that the adaptive immunity mediated by T and B lymphocytes provides a critical foundation for effective and sustained antitumor responses (57).

Above evidences hinted that patients with different PBMC transcriptome subtypes may have different clinical outcomes. However, due to the limitation of small sample size and insufficient clinical data, the direct association between the PBMC subtypes and disease recurrence or cancer survival remains unexplored in our analysis. To partially overcome this, we identified two immune-related gene signatures in PBMCs and examined their power of predicting clinical outcomes using *in silico* prognostic analysis on their expressions in BC tissue samples. Both gene signatures showed the ability to predict the survival of BC patients (Figure 5 and Additional File 7), which is similar to the findings observed by Foulds et al. (14). In their study, they measured PBMC expression values of 800 immune-related genes and investigated their implications on clinical outcomes. They reported that the expression of CD163, CXCR4, and THBS1 in PBMCs could predict the relapse-free survival for triple negative BC patients (14). In our results, the higher expression of signature genes in tumor tissue corresponded to a lower risk of cancer recurrence and better survival rate. Interestingly, the BC patients with subtype_1 might had smaller metastasis probability and better prognosis, because they had higher proportion of lymphocytes, smaller proportion of neutrophils and lower NLR. However, the expression values of the two sets of signature genes were down-regulated in subtype_1. Therefore, we proposed that the up-regulation expression of immune-related genes in peripheral blood is probably related to a down-regulated expression in tumor tissue. This is very similar to the findings in literatures that the regulation of immune-related gene expression is opposite in blood and tissue (58).

## Conclusions

In conclusion, we identified two new subtypes of BC based on their PBMC expression profiles. The two PBMC transcriptome subtypes had distinct immune patterns, which was associated with different immune cell abundances. *In silico* prognostic analysis suggested that BC patients of the two subtypes may have different clinical outcomes. Although this classification is probably useful for personalized BC management, further investigation in a large prospective setting is required to ascertain their clinical values.

## Data Availability Statement

The raw data supporting the conclusions of this manuscript will be made available by the authors, without undue reservation, to any qualified researcher.

## Ethics Statement

The study was approved by the ethical committee of the First Affiliated Hospital of Nanjing Medical University. All samples were used according to the ethical guidelines of the 1975 Declaration of Helsinki and obtained with the patients' understanding that it might be published. The written informed consent was obtained from the participants of this study.

## Author Contributions

This study was conceptualized and designed by WM, XS, HL, YL, and WG. Samples were collected and provided by HX and YZ. RNA sequencing was completed by YB. ZH and YC contributed to the analysis of RNA-seq data and the estimation of the abundance of immune cells. WM completed the discovery and validation of new subtypes, and performed the survival analysis and statistical analysis. The draft manuscript was developed by WM and WG. All authors reviewed the draft and provided comments, contributing to the final version of the manuscript, read and approved the submission and publication.

### Conflict of Interest

The authors declare that the research was conducted in the absence of any commercial or financial relationships that could be construed as a potential conflict of interest.

## References

[1] Fan L Strasser-Weippl K Li JJ St. Louis J Finkelstein DM Yu KD Breast cancer in China. Lancet Oncol. (2014) 15:e279–89. 10.1016/S1470-2045(13)70567-9 24872111

[2] Perou CM Sørlie T Eisen MB van de Rijn M Jeffrey SS Rees CA. Molecular portraits of human breast tumours. Nature. (2000) 406:747–52. 10.1038/35021093 10963602

[3] Sørlie T Perou CM Tibshirani R Aas T Geisler S Johnsen H. Gene expression patterns of breast carcinomas distinguish tumor subclasses with clinical implications. Proc Natl Acad Sci USA. (2001) 98:10869–74. 10.1073/pnas.191367098 11553815 PMC58566

[4] Prat A Parker JS Karginova O Fan C Livasy C Herschkowitz JI. Phenotypic and molecular characterization of the claudin-low intrinsic subtype of breast cancer. Breast Cancer Res. (2010) 12:R68. 10.1186/bcr2635 20813035 PMC3096954

[5] Voduc KD Cheang MCU Tyldesley S Gelmon K Nielsen TO Kennecke H. Breast cancer subtypes and the risk of local and regional relapse. J Clin Oncol. (2010) 28:1684–91. 10.1200/JCO.2009.24.9284 20194857

[6] Hammond MEH Hayes DF Dowsett M Allred DC Hagerty KL Badve S. American Society of Clinical Oncology/College of American Pathologists guideline recommendations for immunohistochemical testing of estrogen and progesterone receptors in breast cancer (unabridged version). Arch Pathol Lab Med. (2010) 134:e48–72. 10.1043/1543-2165-134.7.e48 20586616

[7] Harris LN Ismaila N McShane LM Andre F Collyar DE Gonzalez-Angulo AM. Use of biomarkers to guide decisions on adjuvant systemic therapy for women with early-stage invasive breast cancer: American Society of Clinical Oncology Clinical Practice Guideline. J Clin Oncol. (2016) 34:1134–50. 10.1200/JCO.2015.65.2289 26858339 PMC4933134

[8] Nagarajan D McArdle SEB. Immune landscape of breast cancers. Biomedicines. (2018) 6:20. 10.3390/biomedicines6010020 29439457 PMC5874677

[9] Noy R Jeffrey Pollard W. Tumor-associated macrophages: from mechanisms to therapy. Immunity. (2014) 41:49–61. 10.1016/j.immuni.2014.06.010 25035953 PMC4137410

[10] Talmadge JE Gabrilovich DI. History of myeloid-derived suppressor cells. Nat Rev Cancer. (2013) 13:739. 10.1038/nrc3581 24060865 PMC4358792

[11] Suzuki E Sugimoto M Kawaguchi K Pu F Uozumi R Yamaguchi A. Gene expression profile of peripheral blood mononuclear cells may contribute to the identification and immunological classification of breast cancer patients. Breast Cancer. (2018) 26:282–9. 10.1007/s12282-018-0920-2 30317464

[12] Dumeaux V Ursini-Siegel J Flatberg A Fjosne HE Frantzen JO Holmen MM. Peripheral blood cells inform on the presence of breast cancer: a population-based case-control study. Int J Cancer. (2015) 136:656–67. 10.1002/ijc.29030 24931809 PMC4278533

[13] Hensler M Vančurová I Becht E Palata O Strnad P Tesarová P. Gene expression profiling of circulating tumor cells and peripheral blood mononuclear cells from breast cancer patients. OncoImmunology. (2016) 5:e1102827. 10.1080/2162402X.2015.1102827 27141386 PMC4839342

[14] Foulds GA Vadakekolathu J Abdel-Fatah TMA Nagarajan D Reeder S Johnson C. Immune-phenotyping and transcriptomic profiling of peripheral blood mononuclear cells from patients with breast cancer: identification of a 3 gene signature which predicts relapse of triple negative breast cancer. Front Immunol. (2018) 9:2028. 10.3389/fimmu.2018.02028 30254632 PMC6141692

[15] Baine MJ Chakraborty S Smith LM Mallya K Sasson AR Brand RE. Transcriptional profiling of peripheral blood mononuclear cells in pancreatic cancer patients identifies novel genes with potential diagnostic utility. PLoS ONE. (2011) 6:e17014. 10.1371/journal.pone.0017014 21347333 PMC3037404

[16] Ciarloni L Hosseinian Ehrensberger S Imaizumi N Monnier-Benoit S Nichita C Myung SJ Kim JS. Development and clinical validation of a blood test based on 29-gene expression for early detection of colorectal cancer. Clin Cancer Res. 22:4604–11. (2016) 10.1158/1078-0432.CCR-15-2057 27126992

[17] Brodin P Davis MM. Human immune system variation. Nat Rev. Immunol. (2017) 17:21–9. 10.1038/nri.2016.125 27916977 PMC5328245

[18] Standish LJ Sweet ES Novack J Wenner CA Bridge C Nelson A. Breast cancer and the immune system. J Soc Integr Oncol. (2008) 6:158–68. 10.2310/7200.2008.0027 19134448 PMC2845458

[19] Goldhirsch A Wood WC Coates AS Gelber RD Thürlimann B Senn HJ Panel, strategies for subtypes–dealing with the diversity of breast cancer: highlights of the St. Gallen International Expert Consensus on the Primary Therapy of Early Breast Cancer 2011. Ann Oncol. (2011) 22:1736–47. 10.1093/annonc/mdr304 21709140 PMC3144634

[20] Lappalainen I Almeida-King J Kumanduri V Senf A Spalding JD Ur-Rehman S. The European Genome-phenome Archive of human data consented for biomedical research. Nat Genet. (2015) 47:692–5. 10.1038/ng.3312 26111507 PMC5426533

[21] Dumeaux V Fjukstad B Fjosne HE Frantzen JO Holmen MM Rodegerdts E. Interactions between the tumor and the blood systemic response of breast cancer patients. PLoS Comput Biol. (2017) 13:e1005680. 10.1371/journal.pcbi.1005680 28957325 PMC5619688

[22] Kim D Langmead B Salzberg SL. HISAT: a fast spliced aligner with low memory requirements. Nat Methods. (2015) 12:357–60. 10.1038/nmeth.3317 25751142 PMC4655817

[23] Smyth GK Shi W Liao Y. FeatureCounts: an efficient general purpose program for assigning sequence reads to genomic features. Bioinformatics. (2013) 30:923–30. 10.1093/bioinformatics/btt656 24227677

[24] Pertea M Pertea GM Antonescu CM Chang TC Mendell JT Salzberg SL. StringTie enables improved reconstruction of a transcriptome from RNA-seq reads. Nat Biotechnol. (2015) 33:290–5. 10.1038/nbt.3122 25690850 PMC4643835

[25] Love MI Huber W Anders S. Moderated estimation of fold change and dispersion for RNA-seq data with DESeq2. Genome Biol. (2014) 15:550. 10.1186/s13059-014-0550-8 25516281 PMC4302049

[26] Ritchie ME Smyth GK Phipson B Wu D Hu Y Shi W. limma powers differential expression analyses for RNA-sequencing and microarray studies. Nucleic Acids Res. (2015) 43:e47. 10.1093/nar/gkv007 25605792 PMC4402510

[27] Zhou Y Zhou B Pache L Chang M Khodabakhshi AH Tanaseichuk O. Metascape provides a biologist-oriented resource for the analysis of systems-level datasets. Nat Commun. (2019) 10:1523. 10.1038/s41467-019-09234-6 30944313 PMC6447622

[28] Fabregat A Jupe S Matthews L Sidiropoulos K Gillespie M Garapati P. The reactome pathway knowledgebase. Nucleic Acids Res. (2018) 46:D649–55. 10.1093/nar/gkx1132 29145629 PMC5753187

[29] Monti S Tamayo P Mesirov J Golub T Consensus clustering: a resampling-based method for class discovery and visualization of gene expression microarray data. Mach Learn. (2003) 52:91–118. 10.1023/a:1023949509487

[30] Kapp AV Tibshirani R. Are clusters found in one dataset present in another dataset? Biostatistics. (2006) 8:9–31. 10.1093/biostatistics/kxj029 16613834

[31] R Core Team. R: A Language and Environment for Statistical Computing. Vienna: R Foundation for Statistical Computing (2017).

[32] Newman AM Liu CL Green MR Gentles AJ Feng W Xu Y. Robust enumeration of cell subsets from tissue expression profiles. Nat Methods. (2015) 12:453–7. 10.1038/nmeth.3337 25822800 PMC4739640

[33] Györffy B Lanczky A Eklund AC Denkert C Budczies J Li Q. An online survival analysis tool to rapidly assess the effect of 22,277 genes on breast cancer prognosis using microarray data of 1,809 patients. Breast Cancer Res Treat. (2010) 123:725–31. 10.1007/s10549-009-0674-9 20020197

[34] Koh CH Bhoo-Pathy N Ng KL Jabir RS Tan GH See MH. Utility of pre-treatment neutrophil-lymphocyte ratio and platelet-lymphocyte ratio as prognostic factors in breast cancer. Br J Cancer. (2015) 113:150–8. 10.1038/bjc.2015.183 26022929 PMC4647546

[35] Ethier JL Desautels D Templeton A Shah PS Amir E. Prognostic role of neutrophil-to-lymphocyte ratio in breast cancer: a systematic review and meta-analysis. Breast Cancer Res. (2017) 19:2. 10.1186/s13058-016-0794-1 28057046 PMC5217326

[36] Whyte MKB Walmsley SR Grecian R. The role of neutrophils in cancer. Br Med Bull. (2018) 128:5–14. 10.1093/bmb/ldy029 30137312 PMC6289220

[37] Mishra S Srivastava AK Suman S Kumar V Shukla Y. Circulating miRNAs revealed as surrogate molecular signatures for the early detection of breast cancer. Cancer Lett. (2015) 369:67–75. 10.1016/j.canlet.2015.07.045 26276721

[38] Aarøe J Lindahl T Dumeaux V Saebø S Tobin D Hagen N. Gene expression profiling of peripheral blood cells for early detection of breast cancer. Breast Cancer Res. (2010) 12:R7. 10.1186/bcr2472 20078854 PMC2880427

[39] Baranzini SE Galwey NW Wang J Khankhanian P Lindberg R Pelletier D. Pathway and network-based analysis of genome-wide association studies in multiple sclerosis. Hum Mol Genet. (2009) 18:2078–90. 10.1093/hmg/ddp120 19286671 PMC2678928

[40] Asagiri M Takayanagi H. The molecular understanding of osteoclast differentiation. Bone. (2007) 40:251–64. 10.1016/j.bone.2006.09.023 17098490

[41] Le Pape F Vargas G Clézardin P. The role of osteoclasts in breast cancer bone metastasis. J Bone Oncol. (2016) 5:93–5. 10.1016/j.jbo.2016.02.008 27761364 PMC5063222

[42] David Waning L Khalid Mohammad S Theresa Guise A. Cancer-associated osteoclast differentiation takes a good look in the miR(NA)ror. Cancer Cell. (2013) 24:407–9. 10.1016/j.ccr.2013.10.001 24135278 PMC3897244

[43] Lee M Rhee I. Cytokine signaling in tumor progression. Immune Netw. (2017). 17:214–27. 10.4110/in.2017.17.4.214 28860951 PMC5577299

[44] Grivennikov SI Greten FR Karin M. Immunity, inflammation, and cancer. Cell. (2010) 140:883–99. 10.1016/j.cell.2010.01.025 20303878 PMC2866629

[45] Esquivel-Velázquez M Ostoa-Saloma P Palacios-Arreola MI Nava-Castro KE Castro JI Morales-Montor J. The role of cytokines in breast cancer development and progression. J Interferon Cytokine Res. (2015) 35:1–16. 10.1089/jir.2014.0026 25068787 PMC4291218

[46] Sasser AK Sullivan NJ Studebaker AW Hendey LF Axel AE Hall BM. Interleukin-6 is a potent growth factor for ER-alpha-positive human breast cancer. FASEB J. (2007) 21:3763–70. 10.1096/fj.07-8832com 17586727

[47] Zu X Zhang Q Cao R Liu J Zhong J Wen G Cao D. Transforming growth factor-β signaling in tumor initiation, progression and therapy in breast cancer: an update. Cell Tissue Res. (2012) 347:73–84. 10.1007/s00441-011-1225-3 21845401

[48] Liu ZG. Molecular mechanism of TNF signaling and beyond. Cell Res. (2005) 15:24–7. 10.1038/sj.cr.7290259 15686622

[49] Wu Y Zhou BP TNF-α/NF-kB/Snail pathway in cancer cell migration and invasion. Br J cancer. (2010) 102:639–44. 10.1038/sj.bjc.6605530 20087353 PMC2837572

[50] Wolczyk D Zaremba-Czogalla M Hryniewicz-Jankowska A Tabola R Grabowski K Sikorski AFet al. TNF-α promotes breast cancer cell migration and enhances the concentration of membrane-associated proteases in lipid rafts. Cell Oncol. (2016) 39:353–63. 10.1007/s13402-016-0280-x 27042827 PMC4972855

[51] Lando C Ceppi M Bonassi S Bigatti MP Cebulska-Wasilewska A Fabianova E. An increased micronucleus frequency in peripheral blood lymphocytes predicts the risk of cancer in humans. Carcinogenesis. (2007) 28:625–31. 10.1093/carcin/bgl177 16973674

[52] Ali HR Provenzano E Liu B Dawson SJ Caldas C Rakha E. Association between CD8+ T-cell infiltration and breast cancer survival in 12 439 patients. Ann Oncol. (2014) 25:1536–43. 10.1093/annonc/mdu191 24915873

[53] Wculek SK Malanchi I. Neutrophils support lung colonization of metastasis-initiating breast cancer cells. Nature. (2015) 528:413–7. 10.1038/nature16140 26649828 PMC4700594

[54] Ocana A Nieto-Jiménez C Pandiella A Templeton AJ. Neutrophils in cancer: prognostic role and therapeutic strategies. Mol Cancer. (2017) 16:137. 10.1186/s12943-017-0707-7 28810877 PMC5558711

[55] Azab B Bhatt VR Phookan J Murukutla S Kohn N Terjanian T. Usefulness of the neutrophil-to-lymphocyte ratio in predicting short- and long-term mortality in breast cancer patients. Ann Surg Oncol. (2012) 19:217–24. 10.1245/s10434-011-1814-0 21638095

[56] Jia W Wu J Jia H Yang Y Zhang X Chen K. The peripheral blood neutrophil-to-lymphocyte ratio is superior to the lymphocyte-to-monocyte ratio for predicting the long-term survival of triple-negative breast cancer patients. PLoS ONE. (2015) 10:e0143061. 10.1371/journal.pone.0143061 26580962 PMC4666347

[57] Salgado R Denkert C Demaria S Sirtaine N Klauschen F Pruneri G. The evaluation of tumor-infiltrating lymphocytes (TILs) in breast cancer: recommendations by an International TILs Working Group 2014. Ann Oncol. (2014) 26:259–71. 10.1093/annonc/mdu450 25214542 PMC6267863

[58] Zhang XC Wang J Shao GG Wang Q Qu X Wang B. Comprehensive genomic and immunological characterization of Chinese non-small cell lung cancer patients. Nat Commun. (2019) 10:1772. 10.1038/s41467-019-09762-1 30992440 PMC6467893

