# Supplementary material for: Two Distinct Subtypes Revealed in Blood Transcriptome of Breast Cancer Patients With an Unsupervised Analysis
Source: Front Oncol. 2019 Oct 1;9:985. doi: 10.3389/fonc.2019.00985 (PMC6779774; doi:10.3389/fonc.2019.00985)
Supplement: Supplementary file 1 [file Table_1.DOCX]

**Additional file 1:**

**Table S1.** Demographics of BC patients in the validation cohort.

| **Characteristic** | **All Patients (n = 173)** |
| --- | --- |
| **Age (y)*** | 56.3 (43-104) |
| **Weight (kg)*** | 71.7 (50-150) |
| **Menopausal status**  Premenopausal  Postmenopausal  Not available | 13  138  22 |
| **ER status**  Positive  Negative | 139  34 |
| **HER2 status**  Positive  Negative | 40  133 |
| **PAM50 molecular subtype**  Luminal-A  Luminal-B  HER2-enriched  Basal-like  Normal-like | 48  43  26  29  27 |

Note. Unless otherwise indicated, data are number of patients. *Data for continuous variables are means, with ranges in parentheses.

**
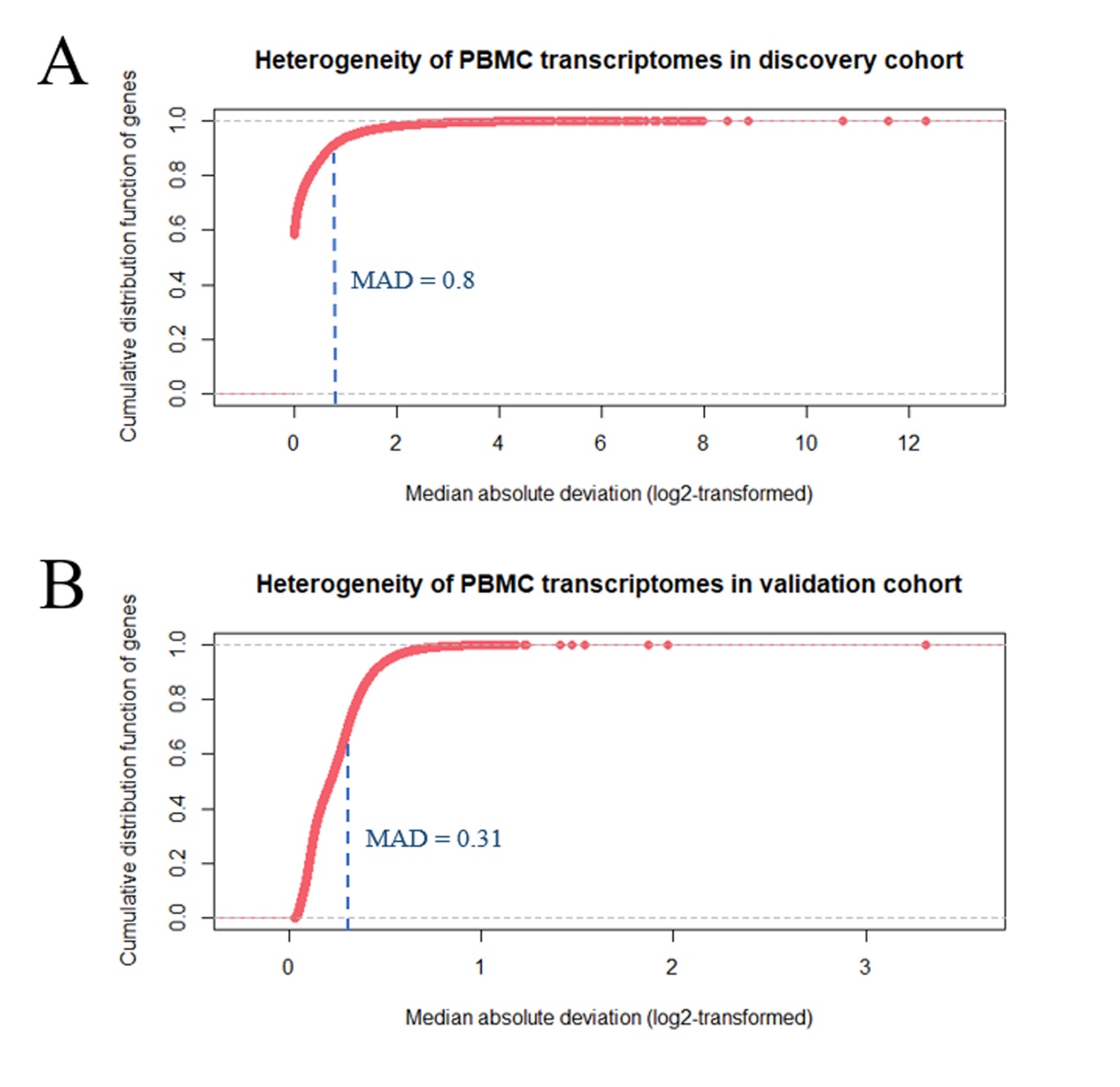
Additional file 2:**

**Figure S1. Heterogeneity of PBMC transcriptome in the BC patients.** Variation of gene expression values among PBMC samples in the discovery (**A**) and validation cohorts (**B**), respectively. X-axis represents the log2-transformed value of median absolute deviation (MAD), and y-axis denotes the cumulative distribution of MAD. The dotted line displays the threshold of log2-transformed MAD for the top 5,000 variable genes.


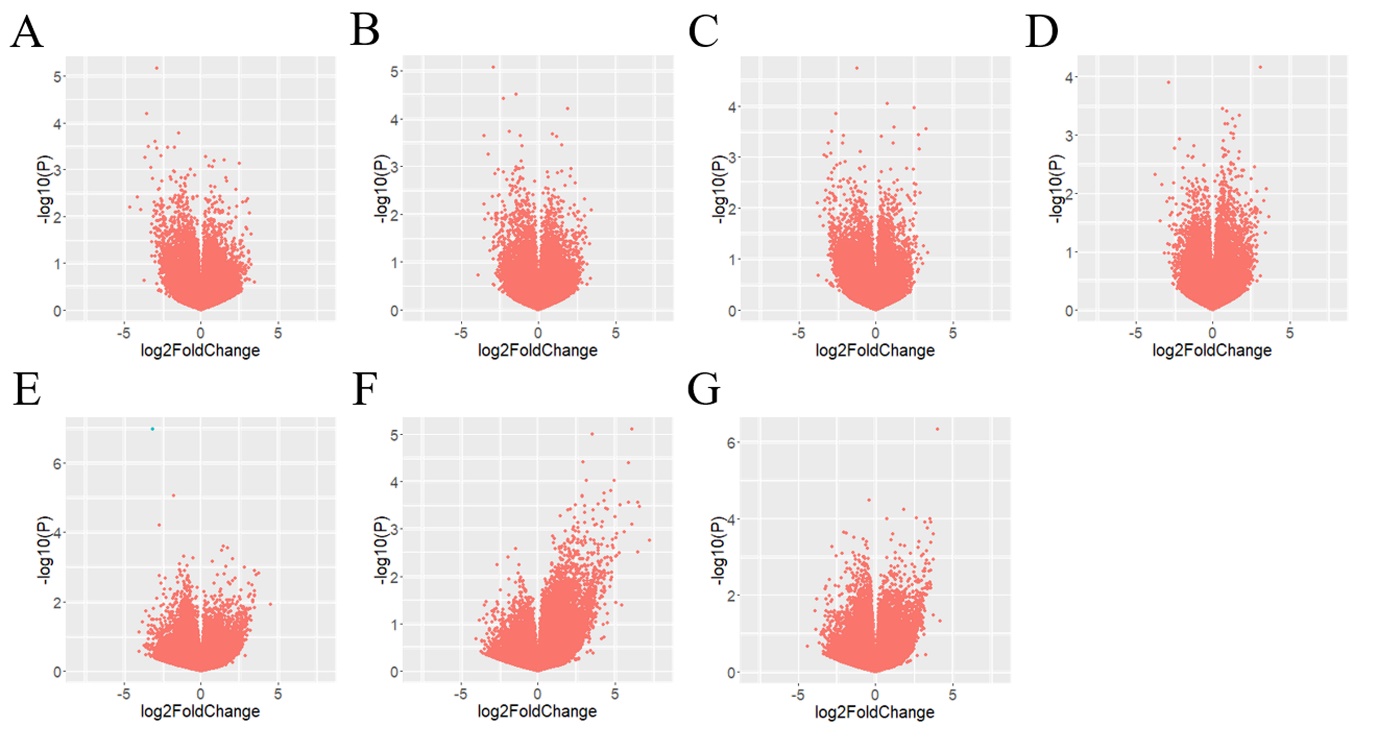
**Additional file 3:**

**Figure S2. Established subtypes of BC showed no difference in PBMC transcriptome in the discovery cohort.** Differential gene expression between ER positive and negative patients (**A**), PR positive and negative patients (**B**), HER2 positive and negative patients (**C**), luminal-A and non-luminal-A patients (**D**), luminal-B and non-luminal-B patients (**E**), HER2-positive and non-HER2-positive patients (**F**), Triple negative and non-Triple negative patients (**G**). These results indicate that established clinical classifications cannot account for PBMC expression heterogeneity in the discovery BC cohort.

**Additional file 4:**
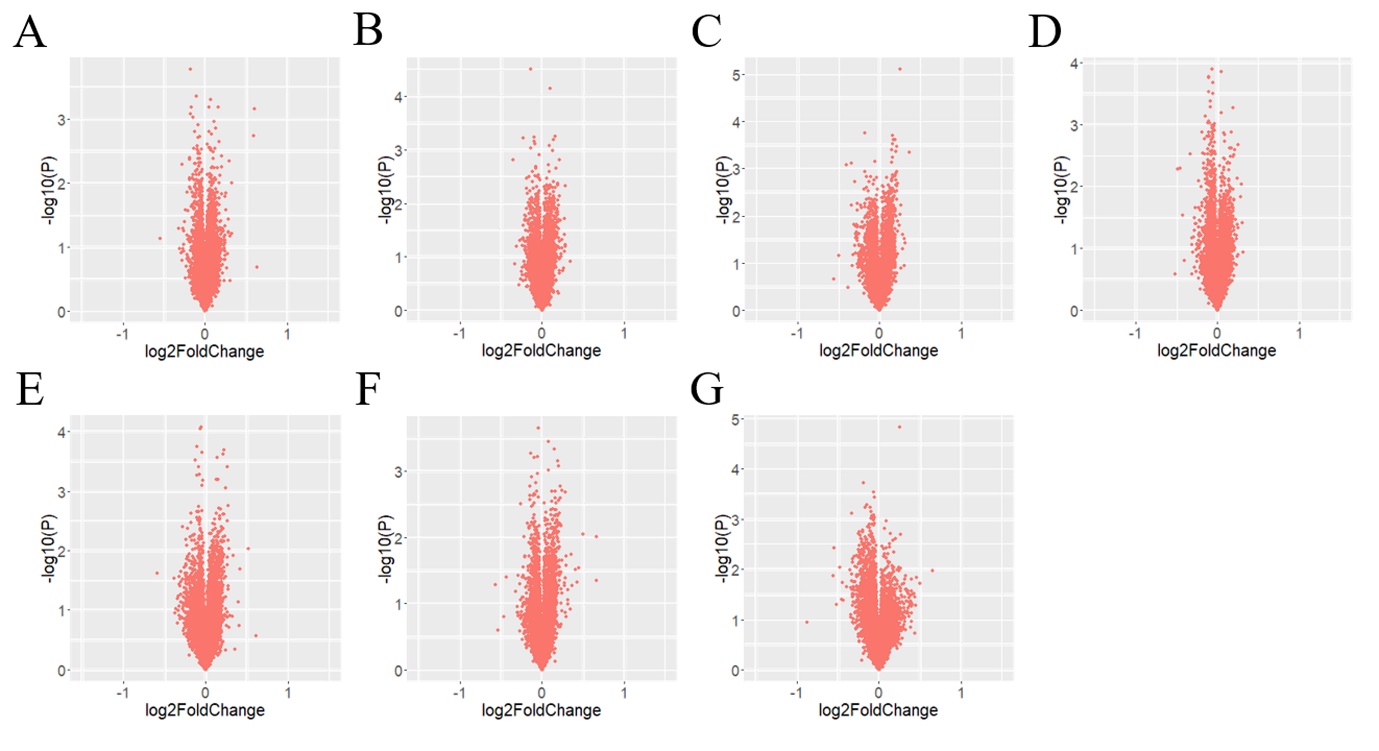


**Figure S3. Established subtypes of BC showed no difference in PBMC transcriptome in the validation cohort.** Differential gene expression of ER positive and negative patients (**A**), HER2 positive and negative patients (**B**), luminal-A and non-luminal-A patients (**C**), luminal-B and non-luminal-B patients (**D**), HER2-enriched and non-HER2-enriched patients (**E**), basal-like and non-basal-like patients (**F**), normal-like and non-normal-like patients (**G**). These results indicate that established clinical classifications cannot account for PBMC expression heterogeneity in the validation cohort.

**Additional file 5:**

**Table S2.** Gene symbols of the 16-gene signature.

|  | **Gene symbol** |
| --- | --- |
| **16-gene signature** | CCL20, IL1A, IL1RN, CCR1, FPR1, IL1R2, LIF, PTAFR, TNFRSF1A, IL1B, ICAM1, IL1R1, CSF1, PTGS2, CXCL1, CXCL8 |

**Additional file 6:**


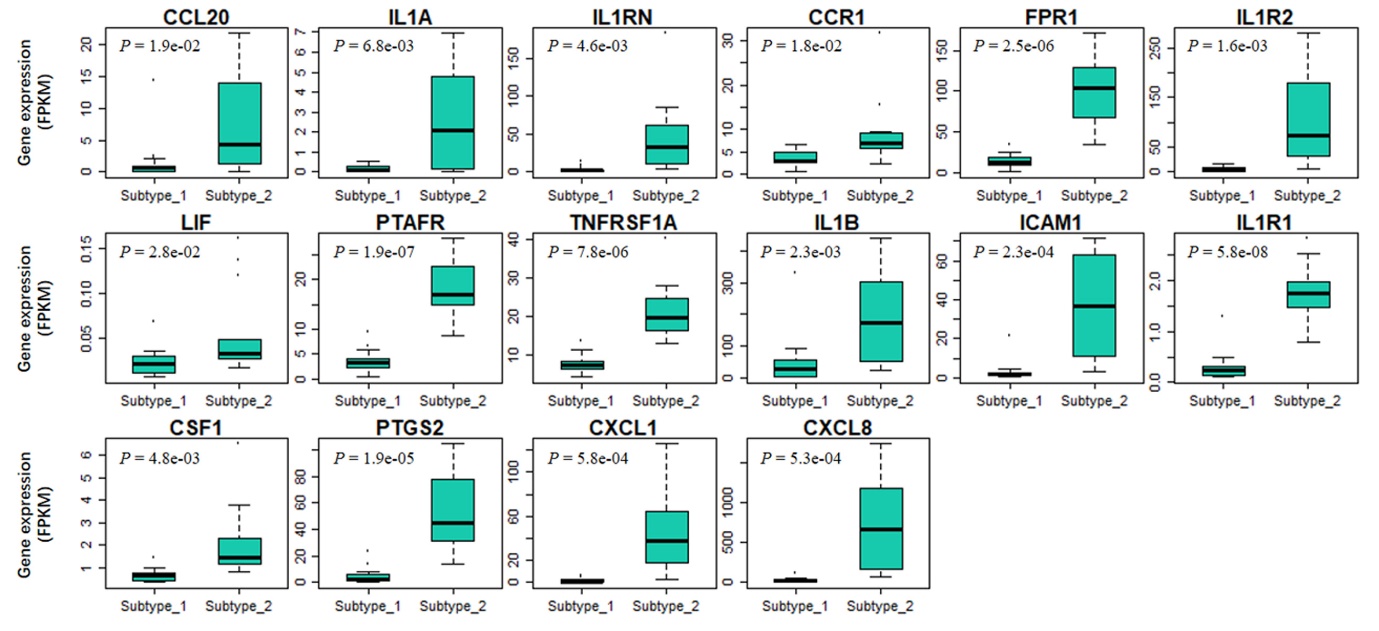


**Figure S4. The 16 immune-related genes differentially expressed in the two PBMC subtypes.** 16-gene signature are derived from the immune-related genes in interleukin-10 signaling pathway. All these genes are lowly expressed in subtype_1.

**Additional file 7:**


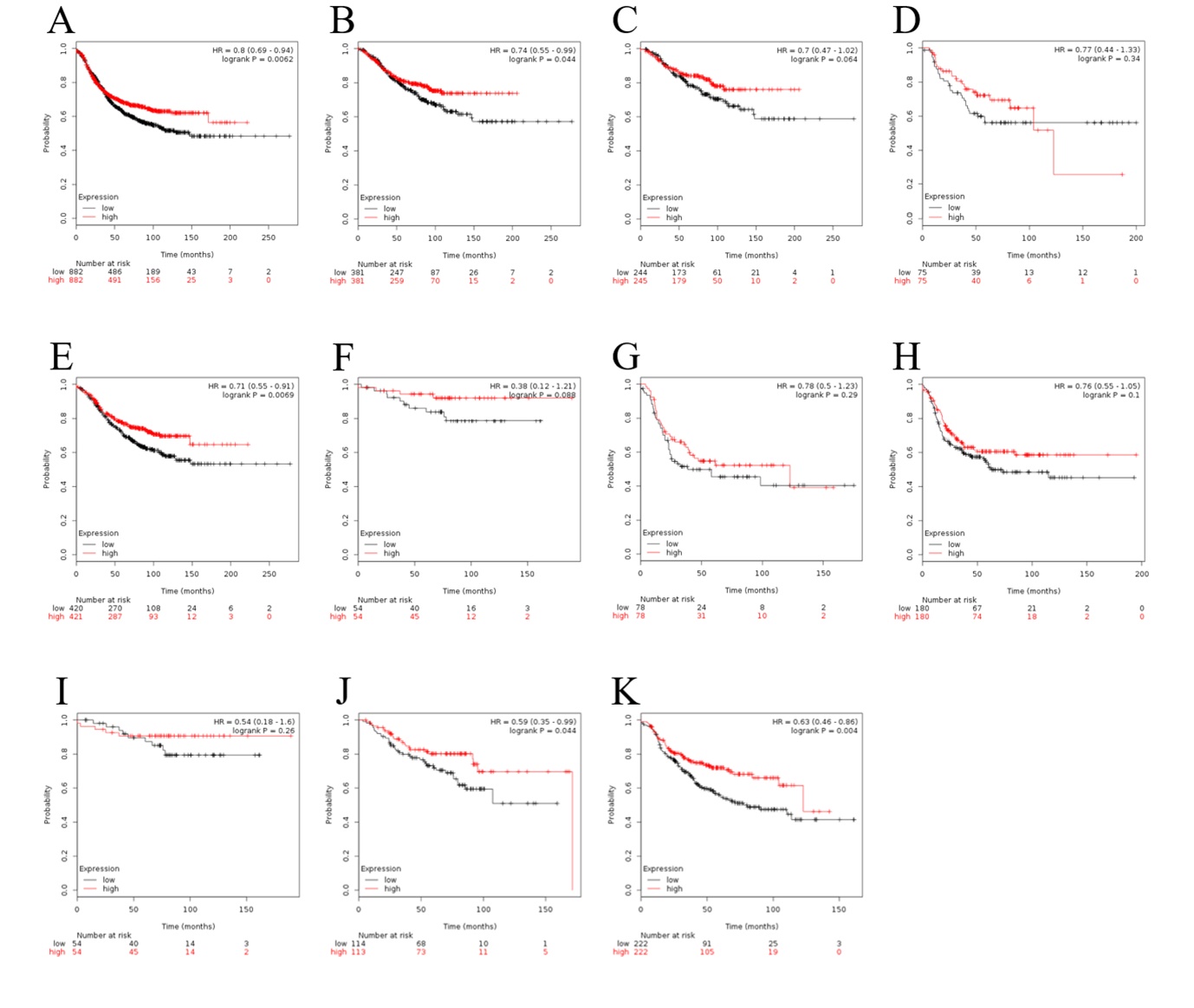


**Figure S5. *Kaplan-Meier* curves of RFS for BC patients with different subtypes and clinical stages by the 16-gene signature.** Prediction result of all subtypes of BC patients (**A**), ER positive patients (**B**), PR positive patients (**C**), HER2 positive patients (**D**), luminal-A patients (**E**), luminal-B patients (**F**), HER2-enriched patients (**G**), basal-like patients (**H**), clinical stage Ⅰ patients (**I**), clinical stage Ⅱ patients (**J**), and clinical stage Ⅲ patients (**K**).

**Additional file 8:**


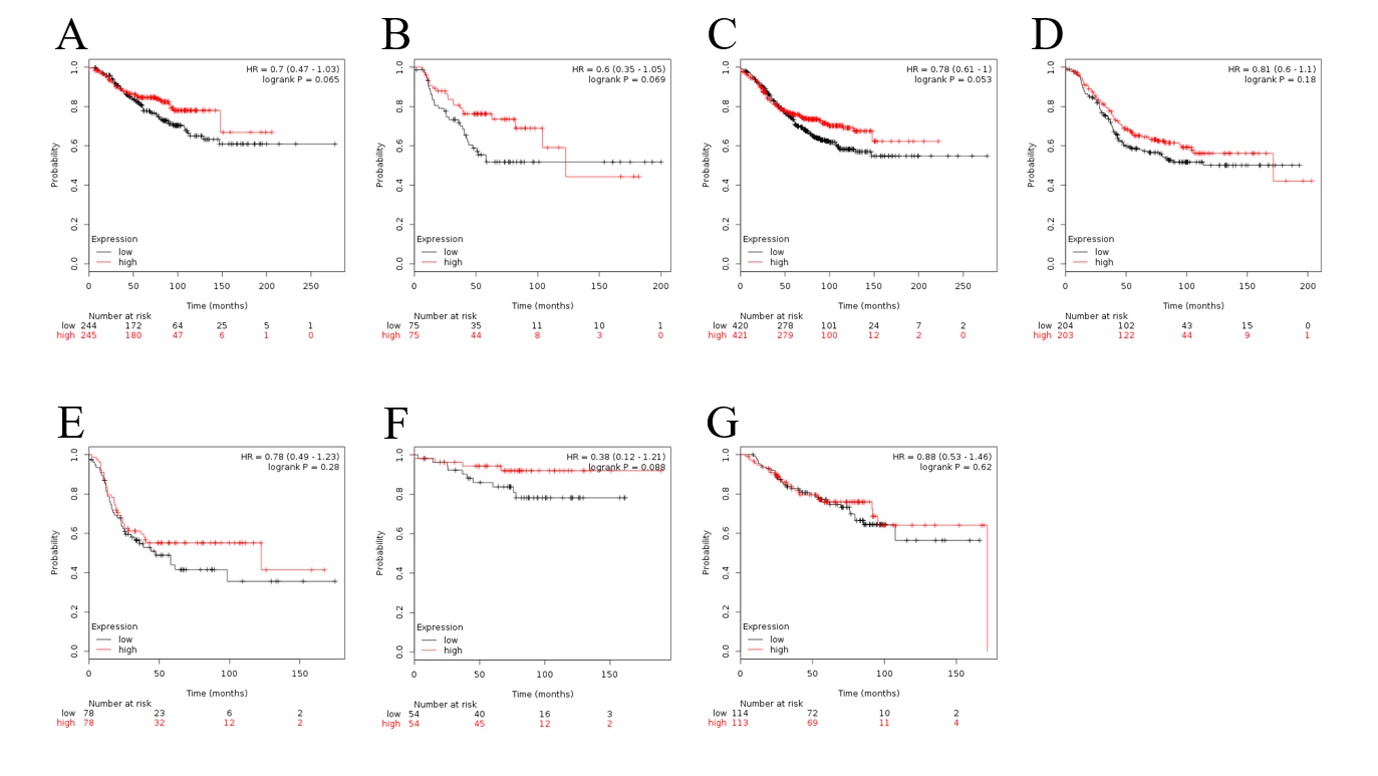


**Figure S6. *Kaplan-Meier* curves of RFS showed no difference in BC patients with PR+, HER2+, luminal-A, luminal-B, HER2-enriched, stage Ⅰ and stage Ⅱ by the 28-gene signature.** Prediction result of PR positive patients (**A**), HER2 positive patients (**B**), luminal-A patients (**C**), luminal-B patients (**D**), HER2-enriched patients (**E**), clinical stage Ⅰ patients (**F**), and clinical stage Ⅱ patients (**G**).
